# Liberal Proposition

**Published:** 1885-11-20

**Authors:** 


### LIBERAL PROPOSITION.

To any one who will send us *two new subscribers*, with Four Dollars remitted, we will send a copy of Hall's "PROBLEM OF HUMAN LIFE"—a wonderful book, now attracting great attention in the scientific and Christain world. The publisher's price for the work is $2. It would make a splendid Christmas or New Year's gift for a friend; one which would do him good morally, and would prove a life-time reminder of your friendship and kindness.

